# Launching Financial Incentives for Physician Groups to Improve Equity of Care by Patient Race and Ethnicity

**DOI:** 10.1111/1468-0009.12720

**Published:** 2024-10-25

**Authors:** HECTOR P. RODRIGUEZ, SARAH D. EPSTEIN, AMANDA L. BREWSTER, TIMOTHY T. BROWN, STACY CHEN, SALMA BIBI

**Affiliations:** ^1^ *School of Public Health* University of California Berkeley

**Keywords:** financial incentives, health equity, quality of care

## Abstract

**Policy Points**

- What are the facilitators and barriers of physician group participation in a performance‐based financial incentive program aimed at improving equity of care by patient race and ethnicity?
- Launching financial incentives to improve racial equity has required extensive organizational change management for participating physician groups, including major investments to improve quality management systems. Carefully designing financial incentives to encourage equity improvement while managing unintended consequences, and considering physician groups’ populations served, baseline maturity of quality management systems, and efforts to assess and address patients’ social risk factors have been central to prepare physician groups for financial incentives to improve equity of care.
- Given the major investments required of physician groups to prepare for financial incentives that reward equity improvement, alignment of equity of care measure specifications and reporting requirements across payers could facilitate physician group engagement. Evidence about how baseline physician group capabilities, including the maturity of their quality management systems, impact equity improvement may help health plans prioritize and target their investments to advance equity of care by patient race and ethnicity.

**Context:**

Blue Cross Blue Shield of Massachusetts (BCBSMA), a large commercial health insurer, is using financial incentives to advance equity of care by patient race and ethnicity. Understanding experiences of this payer and its contracted physician groups can inform efforts elsewhere. We qualitatively assess physician groups’ barriers and facilitators of planning and implementing BCBSMA's financial incentives to improve equity of ambulatory care quality by patient race and ethnicity.

**Methods:**

Key informant interviews (*n* = 44) of the physician group, BCBSMA, and external stakeholders were conducted, equity initiative meetings were observed, and documents were analyzed to identify barriers and facilitators of designing and preparing for financial incentives to advance racial equity. Physician group experiences of preparing for and responding to financial incentives for equity improvement were assessed.

**Findings:**

Analyses revealed 1) the central importance of valid and reliable equity performance measurement and carefully designed equity improvement incentives for physician group buy‐in, 2) that prior to implementing financial incentives for equity improvement, physician groups needed to improve their quality management systems and the accuracy and completeness of patient race and ethnicity data, and 3) physician groups’ populations served, baseline maturity of quality management systems, and efforts to assess and address patients’ social risk factors were central to consider to plan for physician group financial incentives to improve racial equity.

**Conclusions:**

Given the major infrastructure investments and organizational change management resources required of physician groups to participate in a financial incentive program designed to reward equity improvement, alignment of equity measurement and performance requirements across payers would facilitate physician groups’ engagement in efforts to improve quality of care for racial and ethnic minority patients.

Blue cross blue shield of massachusetts (bcbsma), a commercial health insurer operating across Massachusetts, is the first national commercial health plan to launch a comprehensive equity strategy that redesigns financial incentives to improve racial equity in quality of care. To incentivize groups to reduce racial and ethnic disparities in quality of ambulatory care, BCBSMA built on their Alternative Quality Contract (AQC), which incentivized physician groups to simultaneously improve quality and patient care experiences while controlling the rising costs of care. Success of the AQC in slowing the rate of medical spending growth while simultaneously improving quality highlights the potential for performance‐based financial incentives to achieve change.^1^, ^2^ It remains unclear, however, whether performance‐based financial incentives for equity improvement can reduce disparities in quality of care by patient race and ethnicity.

Expanding financial incentives to reward improved equity of care may encourage physician groups to monitor racial and ethnic disparities in quality of care and to implement system‐level and targeted interventions to improve equity. If not carefully designed, however, performance‐based financial incentive programs may result in unintended consequences. These include physician group gaming of performance to achieve expectations, which tends to disproportionately reduce access to care for racial and ethnic minority patients and reallocate resources away from socioeconomically disadvantaged communities.^3^, ^4^, ^5^ Because of historical exclusion and the potential for physician group “gaming” of measurements, well‐intended, equity‐focused interventions can be met with skepticism and mistrust.^6^, ^7^ Consequently, genuine attempts to respond to newly implemented incentives may result in unintended effects depending on incentive design and how these incentives are understood.^8^ To help guide the organization's equity efforts, BCBSMA engaged an independent external expert stakeholder advisor group on all initiatives focused on advancing equity. Stakeholder engagement in the design of equity initiatives helped facilitate contractual agreements with physician groups and was guided by experts with decades of experience tackling health inequities across the state.

Physician group financial incentives for equity improvement were integrated into BCBSMA's incentive formula previously used in AQC contracts to simultaneously encourage improved quality and equity while managing cost growth. Each group's contract was negotiated on its own, without being contingent on other groups’ participation in performance‐based financial incentives for equity. Based on physician group stakeholder feedback, it was determined that 20% of the financial rewards in the incentive formula would be based on equity improvement. The 20% weight for equity improvement is an initial figure and was a starting point for contract negotiations with physician groups in the first cohort of receiving financial incentives for equity improvement.

In addition, multiple design decisions were made in an attempt to equalize the playing field for participating physician groups.^9^ First, the equity incentives reward within‐physician‐group improvement over time rather than basing incentive payments on external performance comparisons. As a result, BCBSMA's equity incentive design addresses a common physician group concern about fairness in pay‐for‐performance programs because comparisons with other groups are not the basis for rewarding equity improvement. Second, BCBSMA's performance‐based financial incentive formula incorporates “equity weights,” a retrospective method that adjusts equity incentive payment such that equity improvements are rewarded more when baseline inequities are larger and/or denominators of racial and ethnic minority patients are larger. Third, to ensure reliable performance measurement, BCBSMA incorporated minimum denominator thresholds for racial/ethnic groups and minimum performance thresholds for quality that were established for each physician group based on their historical data. This design feature aims to discourage cherry‐picking (i.e., removing individuals from certain racial/ethnic minority populations from measure denominators through recoding, not accepting them as patients because of perceptions that their quality of care is more difficult to improve, or creating barriers to prevent them from accessing care).^10^ These actions could result in insufficient denominators that make groups ineligible for equity incentive payments. For groups that were not able to integrate equity incentives because of insufficient denominators for racial and ethnic minority subgroups, BCBSMA still provided the group with other support for advancing equity of care, including payment to offset the costs of participating in the BCBSMA equity improvement collaborative and payment to improve the accuracy and completeness of patient race and ethnicity data.^11^

Many health care organizations have elevated equity as a strategic pillar of organizational performance, but there is limited empirical evidence about the most effective methods to prepare physician groups for financial incentives aimed at improving equity care by patient race and ethnicity.^12^, ^13^ Moreover, the resources needed for physician groups to effectively integrate equity performance measures into their quality management systems and implement equity‐focused interventions remain unclear.^13^, ^14^, ^15^, ^16^ We draw on qualitative analyses of interviews and observations of participating physician group stakeholders, BCBSMA staff, and external stakeholders to identify facilitators and barriers of planning and engaging in a performance‐based financial incentive program designed to improve racial equity while simultaneously improving overall quality of care and controlling cost growth.

## Methods

### Data and Study Design

We conducted a qualitative research study using key informant interviews, meeting observations, and document analyses to investigate key stakeholder experiences of planning for and implementing BCBSMA's financial incentives to advance racial equity. A qualitative approach was selected to allow for a comprehensive understanding of stakeholders’ experiences. Between October 12, 2022, and March 16, 2023, 60 key stakeholders were invited to participate. A total of 42 interviews with 44 key stakeholders were conducted for a response rate of 73%. Key stakeholders included 11 physician group leaders, seven Institute for Healthcare Improvement (IHI) staff, 17 BCBSMA staff, five independent external expert stakeholder advisor group members, and four external government stakeholders. The IHI provided technical assistance to groups in the form of a learning collaborative to support groups as they tested interventions to improve equity of care on prioritized measures. External governmental stakeholders were knowledgeable about equity of care initiatives in the public sector and/or were involved with the development of equity standards within the state of Massachusetts.

Interview questions addressed participants’ understanding of the design of financial incentives to advance equity, alignment with other strategic goals of participating physician groups, external stakeholder engagement, and early perceptions of sustainability (see Supporting Information). Of BCBSMA's 14 contracted physician groups, eight were eligible for performance‐based financial incentives for equity of care based on minimum denominators for racial and ethnic groups. At the time of interviews, of the eight eligible groups, five had equity incentives integrated into their 2023 contracts and three were in the process of planning or negotiating equity incentives for inclusion within future contracts. All physician groups had received pilot project grant funding, were actively participating in BCBSMA's equity improvement learning collaborative, and were aware of their eligibility for equity incentives based on minimum subgroup denominators. As such, most physician group stakeholders were aware of whether equity incentives would be integrated into their organization's contractual agreements with BCBSMA. Participants reviewed a consent document before their 30‐ to 60‐minute recorded interviews, which were transcribed and coded after deidentification.

We also observed bimonthly external stakeholder advisor group meetings, monthly quality improvement collaborative sessions, and ad hoc meetings that addressed priority implementation issues, including race, ethnicity, and language data collection and analysis, equity‐focused interventions, and quality improvement methods. Meeting observation notes and quarterly physician group progress reports for BCBSMA‐supported equity improvement initiatives were analyzed with the coded interview data.

### Analysis

Guided by past research on strategies to advance health equity, a codebook of 11 codes and 16 subcodes was iteratively developed by four researchers using deductive and inductive methods.^17^, ^18^, ^19^ We identified and analyzed primary themes reported by stakeholders regarding financial incentives to advance equity, including equity measurement, organizational investments in monitoring and managing equity performance, BCBSMA initiative alignment with other strategic goals of participating physician groups, and the role of community‐based partnerships to help with designing and implementing equity‐focused interventions to meet performance expectations. Four researchers used NVivo, LLC, software to code and analyze the transcripts. To ensure reliability, multiple researchers coded ten interviews (∼20%) spanning the stakeholder groups, which were reviewed by the researchers to reach consensus. Once the research team calibrated coding practices, the remaining transcripts were then each coded by one of the four researchers. NVivo analysis features were used to examine all text segments associated with the focal codes, and themes were documented with supporting text and quotes.

During weekly research team meetings, coding practices were discussed and reconciled, and emergent themes were identified based on discussion. Investigators compared the key informant interview data with meeting documents and field notes to enhance interpretation of the qualitative data. Notes and physician group equity initiative progress reports to BCBSMA were also reviewed along with transcripts summarized by the physician group to help fill in contextual information about each group's equity initiative milestones.

## Results

As BCBSMA designed and began to implement its financial incentives to advance equity of care by patient race and ethnicity, key measurement and incentive design considerations included how to measure equity of care and then how to incentivize it and incorporate equity performance expectations into physician group contracts while also safeguarding against unintended consequences.

Our analyses revealed important facilitators to physician group engagement in BCBSMA's equity incentive program, including confidence that equity performance measures were valid and reliable, leveraging an established performance‐based financial incentive infrastructure (the AQC), and incentive design decisions that facilitated physician group buy‐in. Although physician group stakeholders were generally supportive of the financial incentives for equity improvement, they perceived and experienced misalignment of equity initiative expectations across payers and had concerns that social risk factors such as food insecurity, transportation, and housing instability would impede equity improvement and impact their incentive payment.

Given that the equity incentives were negotiated as part of each group's overall contract, the contract negotiations were high stakes for the groups. BCBSMA leadership reported that no physician group went too far down a path of trying to exclude equity from incentive criteria. Nonparticipation in the equity component for groups eligible to do so based on minimum patient denominators, however, could mean their complete exclusion from BCBSMA's network.

The interviews illustrated that there were important reasons behind the complex equity incentive design but that those complexities meant that some groups misunderstood the details. These overall themes are summarized in Table 1.

**Table 1:** Physician Group Experiences of Preparing for Financial Incentives to Advance Racial Equity

Valid and reliable equity performance measurement	Deciding how to define and measure equity improvement (as distinguished from overall, aggregated quality improvement) is an important first step to elevate equity as part of performance management systems. Equity measurement and incentive design choices that guard against physician group “gaming” and other unintended consequences are critical facilitators of physician group buy‐in for financial incentives to advance equity. Imputation methods and efforts to improve the accuracy and completeness of self‐reported race and ethnicity data both hold promise, but data limitations present barriers to physician group action.
Equity incentive design and alignment with other payers	Leveraging existing value‐based payment infrastructure improves the ease of equity incentive implementation. “Upside only” risk incentive arrangements and other design considerations for equity performance facilitated physician group buy‐in. Cross‐payer alignment, for example between BCBSMA's and Massachusetts's Medicaid programs, facilitated the development and implementation of physician group interventions to address racial inequities in quality of care. BCBSMA's equity initiatives are already having positive spillover effects for other payers (BCBSMA's competitors in many instances). BCBSMA support for payer‐agnostic interventions was a key facilitator of physician group buy‐in and was considered crucial for advancing equity objectives.
Considering physician groups’ populations served, data and improvement infrastructure, and social risk factors	Physician groups who include safety‐net organizations reported uncertainties in advancing racial equity because social risk factors, including food insecurity, housing instability, and limited transportation, may impede equity improvement. Some physician group stakeholders believed that improving equity for patients with elevated social risk factors requires more resources than improving equity for patients with fewer social risk factors. Safety‐net physician group stakeholders are uncertain about their rate of equity improvement given barriers presented by social risk factors.

BCBSMA, Blue Cross Blue Shield of Massachusetts.

### Valid and Reliable Equity Performance Measurement

For BCBSMA, deciding how to define and measure equity improvement, as distinguished from quality improvement, was an important first step toward integrating equity measures as part of quality management systems. As a BCBSMA stakeholder emphasized, interventions that improve overall quality can still perpetuate inequities or exacerbate inequality by improving quality more for previously advantaged racial/ethnic groups, thus reenforcing underlying disparities: “If an initiative or intervention improves quality across the board, so everyone's quality improves regardless of race or ethnicity, is that health equity? [For certain racial/ethnic groups]… yes, they're better off than they started, but the gap between them and the baseline comparison group is the same or bigger,” (stakeholder 12a).

Table 2 includes additional supporting quotes related to BCBSMA efforts to ensure valid and reliable equity performance measurement. BCBSMA explicitly designed their financial incentives to advance equity with safeguards to discourage performance changes that could lead to declines in quality for any patient group. In theory, a physician group could narrow disparities by reducing quality for a subgroup whose quality had been relatively better, rather than by improving quality for a subgroup whose quality had been relatively worse—for example, narrowing racial disparities by stopping hemoglobin A1c testing for White patients with diabetes. To guard against this possibility, BCBSMA designed financial incentives for equity improvement with provisions that prevent physician groups from earning financial rewards for improving equity at the expense of overall quality of care.

**Table 2:** Ensuring Valid and Reliable Equity Performance Measurement

Deciding how to define and measure equity improvement (as distinguished from quality) is an important first step to elevate equity as part of performance management systems.
Evidentiary Basis (Exemplar Quote[s])
Stakeholder 12a (BCBSMA): “I was surprised when I joined this work how many definitions of health equity there were and how some… seemed really distant from what I thought of as health equity. Some really focused on creating… equitable opportunities for access. And I was like, how about outcomes? […] I was really interested in getting past process and more towards outcomes definitions.”
Stakeholder 8 (BCBSMA): “[…] How exactly are we measuring equity? […] In retrospect it seems almost… obvious where we landed, but I remember… when we first sat down and said… we want to develop a zero to five score to capture equity. […] How exactly to do that was… really amorphous to us…. […] When we say we want to measure equity…what exactly are we measuring? … […] That's one bucket of questions. And then the other bucket of questions is how do you then financially incentivize that and incorporate it into your contracts?”
2. Equity measurement and incentive design choices that guard against physician group “gaming” and other unintended consequences are critical facilitators of physician group buy‐in for financial incentives to advance equity.
Stakeholder 6 (BCBSMA): “[…] There is a floor on how much your denominator size for each racial or ethnic group can change. Because if a group were to drop a specific racial or ethnic group just to make themselves look better and earn more money, that would definitely not be ideal….”
3. Imputation methods and efforts to improve the accuracy and completeness of self‐reported race and ethnicity data both hold promise, but data limitations present barriers to physician group action.
Stakeholder 23 (physician group): “[…] The…challenge we keep having…is…[that] health plans have very limited self‐reported…data. It's all imputed… So…we've had a number of conversations with… [the] Blue Cross team that, hey, we have better data. Why don't we give you the [patient race and ethnicity] data rather than your imputation? And…then that way we can be more confident about the dashboards you've given us. Because the dashboards you're giving us, especially if you're going to start paying us for equity, need to tie to interventions… I get the point; it's not waiting for data perfection. But… once this starts to become high stakes… I think it's important that the data be as reliable as possible.”
Stakeholder 24 (physician group): “[…] This is preliminary data… but I believe there's something on the order of a 20 to 30% mismatch [between provider group internal self‐reported data and BCBSMA‐provided imputed data] for Hispanic and a 49% mismatch for Black patients… So either… from the imputed data, [the] patient is assigned Hispanic… and…we have… another race/ethnicity or… we have Hispanic and the imputation assigns another race or ethnicity to that patient. So they're not insignificant… We're going to have to track our internal performance using our self‐reported^a^ data, and then we're going to have to track how Blue Cross is measuring us on our performance.”
Stakeholder 24 (physician group): “[…] One of the challenges… is that Blue Cross's current approach is to use imputed data…. […] I think from a provider organization perspective, the preference is always going to be to use patient self‐reported data… […] When we track our own internal performance with our physicians, they're going to want to see their self‐reported data,^a^ which will not match a hundred percent with how Blue Cross measures us on actual… improvement of closing disparity gaps… So I see that as a possible issue….”

BCBSMA, Blue Cross Blue Shield of Massachusetts.

^a^ Physician group stakeholders sometimes refer to their own internal race and ethnicity data as “self‐reported” when the data are not fully self‐reported, and some data are administratively determined.

As part of their equity improvement efforts, BCBSMA made major improvements to the accuracy and completeness of their members’ self‐reported race and ethnicity data and uses these self‐reported data combined with imputation methods for equity performance measurement. BCBSMA provides physician groups with retrospective quality‐of‐care data stratified by patient race and ethnicity on an annual basis, which are reportedly very helpful for many groups. Although physician groups found these annual reports helpful, discrepancies between imputed race and ethnicity data and internal race and ethnicity data from physician group information systems were documented by at least one physician group. That physician group stakeholder noted:

[…] This is preliminary data… but I believe there's something on the order of a 20% to 30% mismatch [between physician group internal self‐reported data and BCBSMA‐provided imputed data] for Hispanic and a 49% mismatch for Black patients… We're going to have to track our internal performance using our self‐reported data, and then we're going to have to track how Blue Cross is measuring us on our performance. (Stakeholder 24)

Planning for BCBSMA's financial incentives to advance equity required physician groups to improve their quality management systems, which sometimes did not align with the Healthcare Effectiveness Data and Information Set (HEDIS) measure specifications used by BCBSMA. Physician groups sometimes used “homegrown” quality measures, which were not necessarily consistent with HEDIS. For example, although HEDIS defines achieving hypertension control as having a blood pressure of less than 140/90 mm Hg, at least one group defined achieving hypertension control as the patient having been prescribed three or more antihypertensive medications or as having had one blood pressure check within the past 6 months. Black patients are disproportionately likely to have uncontrolled hypertension even after having been prescribed three antihypertensive medications, so this homegrown measure had the effect of masking racial inequities.^20^ BCBSMA worked closely with the physician group to help align internal reporting specifications to BCBSMA's incentive program requirements.

### Design of the Financial Incentives for Equity Improvement

Table 3 summarizes supporting quotes related to the design of financial incentives for equity improvement and alignment of equity initiative requirements with other payers. BCBSMA's AQC experience helped inform decisions about how to measure equity and design incentives for improving equity.^2^ Leveraging the AQC structure enabled the equity component of the incentive formula to be integrated in physician group contracts in a straightforward way. As a BCBSMA stakeholder noted:

**Table 3:** Equity Incentive Design and Alignment With Other Payers

Leveraging existing value‐based payment infrastructure improves the ease of equity incentive implementation.
Stakeholder 12 (BCBSMA): “[…]10 years ago… we started the Alternative Quality Contract … […] So when we started that, that was a major change… Because now providers were at risk and some of their… dollars were tied to… performance and… they were compared to the network. […] That…was, I would say…the major, major, major change… And so they've all adjusted to that now… […] Then equity came along more recently and that fits into the Alternative Quality Contract. So it's not like another totally different program. […] We… look at the quality… scores that are already part of the Alternative Quality Contract, but then we look at it by race.”
Stakeholder 14 (BCBSMA): “[…] Our standard kind of AQC agreement… is primarily weighted on physician, on ambulatory measurement. And then we have a smaller portion tied to hospital measurement. […] That's the way it's always been. And then we added in equity…. And so we really brought it into the same quality calculations that we've been doing since the beginning of AQC. So they'll get an AIM score, which is how they're performing …. […] HIMs, which is Hospital Incentive Measures, and EIMs are Equity Incentive Measures… […] They're also responsible for total medical expense.”
Stakeholder 8 (BCBSMA): [Regarding “key…modifications to…existing contracts as part of…pay‐for‐equity”]: “[…] Our boiler‐plate intent was that we added equity at renewal time as part of the overall negotiation… And… we wanted… for ease of provider adoption and understanding… to insert that into the existing chassis and [in] as seamless of a way as… possible… […] How do you develop an equity score from zero to five, but then once you have that equity score from zero to five, everything from there should actually feel exactly the same to a provider? […] All we did was take that equity score (zero to five), and then blend that into the quality score with a new weighting… And then everything downstream from there should be seamless to the provider's understanding.”
Stakeholder 8 (BCBSMA): “Our very first contracts were actually a hundred percent risk share unlimited up and down risk… And… then we've evolved a bit to a more shared risk model. So the efficiency risk share for most contracts varies between 30 and 70%. And then there's overall aggregate limits of about… $25 to $30 PMPM …. […] In the efficiency measurement the providers are taking risk on total cost of care. […] So we'll mutually agree on methodology to develop a budget. […] Their budget could be something like $500 PMPM and then… we retrospectively looked to see where their actual claims came in. And if their claims came in at like $450, then that's $50 of gross surplus. And then the…30 to 70 [%] that I was mentioning, that's the risk share. So if they have 70% risk share, then after all is said and done, they would get [a] $35 PMPM efficiency payment.”
2. “Upside only” risk incentive arrangements and other design considerations for equity performance facilitated physician group buy‐in.
Stakeholder 3 (BCBSMA): “We [had] brief discussions [about downside risk]. Discussions that lasted only as long as it took for people to say we're not going to do downside risk on this. It's… all upside risk… Honestly it was a pretty short conversation, but a lot of it is around… the behavioral economics associated with upside versus downside risk, and where we are in this and the journey to close the gap. We don't want to be punitive; we want to create an incentive. There's investments required to make this change. So punishing people for trying but not succeeding at it… doesn't feel like the right approach here.”
Stakeholder 25 (physician group): “… It is all feeling quite new, and what I think is going to be a shock to the system is any downside risk…. We're used to upside…. […] In Blue Cross there is a… modifier that relates. Not only do we get a per member per month sort of incentive payment that's very, you know, you get it or you don't, there's a maximum kind of thing. But on the surplus deficit calculation, there is a… modifier that currently is based on the quality score. So if… and when that is, either there's a second modifier that's self‐equity that will become very real… and I would just say we're not, haven't really done those equations yet.”
Stakeholder 1 (BCBSMA): “[…] The other important thing [about equity improvement] is everyone is just being compared with themselves. For this, we're just saying you need to improve on your [equity] performance year over year.”
3. Cross‐payer alignment, for example, between BCBSMA's and Massachusetts's Medicaid programs, facilitated the development and implementation of physician group interventions to address racial inequities in quality of care.
Stakeholder 23 (physician group): “[…] The waiver, for health equity…. […] is relative, but it's $350 million annually across all the ACOs that are participating in the Medicaid program here in Massachusetts…There's… a lot of details pending, but there's three major domains…. The first… is on data completeness, both… REaL data, SO/GI [data], etc. .… […] There's also SDOH screening that's part of the data completeness and does a quarter of the weight. The… second domain is about actual interventions… first related to doing the disparity analysis… and then interventions to close gaps that are found from the disparity analysis. […] That's 50% weight. And then the… third domain has to do with collaboration between providers and in the ACO program… [… and] a requirement to be accredited by the Joint Commission [JCAHO] on the health equity requirements that they just published. […] The third domain…is the… other quarter of the weight, so it's pretty significant.”
Stakeholder 25 (physician group): “[…] If there's not alignment between the different contracts and initiatives, we are just hamstrung….”
Stakeholder 11 (BCBSMA): “[…] One group…. […] opted not to participate in the small group Equity Action Community. And their rationale is like: well we want to wait and hear what MassHealth is doing, because we have a large population of that, and we don't want to start something with you and then have to shift it or change it based on what MassHealth requirements are going to be… […] It's unfortunate… like they get it… they're leaving money on the table. But for them… they see it as the right business call.”
Stakeholder 23 (physician group): “[…] Blue Cross is much more advanced in… their approach… But then… come[s] our state's Medicaid program with… really game changing financial influx of funds into the… new waiver with the feds that just got approved by the state…. So now Medicaid, which is a pretty large payer for us, is putting… national leading amounts of money into… the Medicaid program… specifically related to health equity. So… we're very thankful that we got started with this Blue Cross work, because it's… seeded a lot of the work that… now we have to do at greater scale with even bigger carrots.”
Stakeholder 42 (physician group): “[…] For the MassHealth contracts in particular… those are the biggest dollars we're seeing for sure for the hospital contract. […] Statewide for the hospitals, Mass Health is investing $350 million in health equity… And that shakes out too somewhere between X and Y million for us in one year. […] You know, I don't even know that I have… a number for Blue Cross, but I think it's way smaller.” [amounts redacted for confidentiality]
Stakeholder 42 (physician group): “[…] MassHealth is on the forefront for us [insofar as] … we will go live with incentives for health equity with them first starting in 2023… […] Largely what we're seeing is that in the first few years of the [MassHealth] program, we'll see… pay for reporting… and then certain quality measures disaggregated by race, ethnicity, etc. And then over time they'll start to introduce true accountability for reducing disparities… So I think to that end, pretty similar to what we expect with Blue Cross of Mass [BCBSMA]. There's other requirements that we are seeing related to equity… They require NCQA health equity accreditation. […] It's… a lot of work. But… I think it's pretty amazing how it's accelerating health systems…. […] I will be honest…I am positive that we would not be having these conversations if not for payers’… [and] Massachusetts's pushing us in this direction. I think we would've gotten there eventually, but not at this pace.”
Stakeholder 19 (external stakeholder): “I think in the commercial market, which is really focused on meeting the needs of large, self‐insured employers, I don't think there's going to be a big health equity push from those employers. […] There will be exceptions, but… by and large, no. […] I think if it happens, it's going to be because the accrediting body and NCQA pushes them to do it.”
Stakeholder 25 (physician group): “I think it's if anything… there's the JCAHO and… it's great, because… I think it feels to me like the ball is finally rolling… into one‐ish ball…. […] It doesn't need to be centralized, but it just needs a little more being in the same space… [so that we] take less time to… navigate how should we do this, and who do we need to ask, and what are they all doing anyway kind of thing.”
Stakeholder 25 (physician group): “[…] I personally have some worry of… getting to 80% complete[ness] on race/ethnicity [data]… you really have to get very concrete that this is a… [requirement], and here's the workflow, and get buy‐in at the highest level to do it. […] It has only recently gotten to that highest… level, and honestly, it's from the JCAHO requirement, [which] is what just kicked us going…. So that I think… might end up being part of our catalyst is [that]… we have this JCAHO requirement… and the Medicaid contract, the MassHealth Hospital contract all now have equity, and then we know Blue Cross will too, and so… they align.”
Stakeholder 42 (physician group): “[…] We're not doing any exchange of clinical data that would include demographics data with our affiliates at this time. Community connect will solve that… […] Currently we only have to report certain data to CMS for 70% of our ACO population. They're moving to a hundred percent, which means that we'll need to somehow ingest all of that clinical data from our affiliates and be able to report it back out to CMS. […] That's… complicated but good for what we are talking about, because at some point we're going to have to have bidirectional clinical data exchange… [and] that will support equity work, because then we'll have a fuller picture of the population.”
4. BCBSMA's equity initiatives are already having positive spillover effects for other payers (BCBSMA's competitors in many instances).
Stakeholder 12 (BCBSMA): “There were a lot of providers… […] Always with the state, their questions were more like: how much money are you going to allocate towards this? Because that's a pretty big population for… some of these groups, some more than others, but [for] some of them, it's a lot of their business… and Medicaid already, the rates are pretty low. And so… now to add this on to where… they're like…you are going to make us do a lot more work, but you're not… really paying us that much already. They appreciated [that] Blue Cross had given all this money… but… I think… they felt like they needed more than they thought the state was going to be able to give for this.”
Stakeholder 44 (physician group): “We're leveraging what we're doing… with Blue Cross Blue Shield to achieve our contract requirements for MassHealth… […] They require us to improve our collection of data… to 90% completion… So… we're going to leverage the Blue Cross Blue Shield grant and expertise to help us achieve that… They're also asking us to begin collection of disability and SO/GI data… Again, we'll do that. They expect us to stratify the data by these social risk factors across quality measures… They're also asking us to… come up with innovations for closing the gaps… in health inequities… [and MassHealth is requiring us to] … acquire NCQA accreditation, which requires fulfilling several standards. […] Those standards, again, are part of this health equity capability or infrastructure that we're trying to build with the Blue Cross Blue Shield funding… We can't do this work unless there's upfront investments… because you know, the health system was not built with health equity… in mind… Asking providers to just change how they collected their [data], even just reconfiguring their EHR to ingest this data, requires resources. And if we can't provide these resources, then we should not expect the change anytime soon.”
Stakeholder 44 (physician group): “There are other ACOs… […] I think there are about five of us [small provider groups] that are participating in the Blue Cross Blue Shield [equity initiative]…[…] And I think all of them are receiving Medicaid MassHealth contracts… […] The ACO that I was speaking with this morning, they're treating the accreditation program as… [an] initiative separate from… the other MassHealth requirements and… other payers’ requirements. And to me it seemed like a really missed opportunity. What we've decided to do is bring all these requirements in one place.… So for care management, for example, I look at what the MassHealth contract requires of them. I look at what the Blue Cross contract requires of them, and I look at… what accreditation requirements are, and then I put them together and give them a package of requirements for which they must build capabilities… And if we have the Blue Cross funding… I can say: here's a pot of money that you can use to build this capability. It's going to fulfill our requirements once we're done.”
Stakeholder 28 (physician group): [With respect to BCBSMA]: “… I don't think they realized, but when they designed their AQC contracts… a decade ago and they put a significant amount of funding towards quality…. […] we focused… almost exclusively on quality outcomes for Blue Cross patients. And so… that, in and of itself, created inequity…. […] At the time that they designed [the] AQC, it was with the best intentions of improving quality for their Blue Cross members, but… for provider groups that take multiple insurances and are in a variety of risk contracts, it took all the focus on quality measurement and directed it towards Blue Cross….”
Stakeholder 15 (BCBSMA): “In our initial rollout of the AQC, I think it was very important that… we were very open to tweaks and feedbacks and learning and… the model now bears… little resemblance, except at its core, to the model in 2010.”
Stakeholder 23 (physician group): “We said…. […] our approach will be payer agnostic. We're…not just going to take the 2 million and ask the practices to only focus on Blue Cross patients. That would be completely against the whole concept of equity. And they were very much in agreement with that… […] because there's going to be a… sort of a general interest in, well, let me work on that Blue Cross patient roster first.”
Stakeholder 24 (physician group): “[…] We have decided that we're not going to [home] in on a particular payer because that's not how… physicians or health systems work, right? … […] If there's one thing that… physicians really don't respond to, it's… focus on your Blue Cross patients… So obviously we're going to be tracking both an all population… [and] also an only Blue Cross population… […] but the interventions are not payer specific.”
Stakeholder 28 (physician group): “[…] We're not really paying attention to insurance. This for us is going to be an initiative that's more… ultimately are they a[n] [interviewee's organization] … patient or not? I mean… that's the line we have to draw from a compliance and risk standpoint…. […] We're using this funding for really anybody who qualifies. They're… definitely not going to be just Blue Cross patients. And, quite frankly, our mix of… racial and ethnic minorities are not in Blue Cross… they're in Medicaid…. So, by default there will be patients in different non‐Blue Cross populations that we're doing this work with.”
Stakeholder 40 (physician group): “[…] We understand this is a Blue Cross sponsored grant, however, if we're just focusing on a commercial population, we're missing such a significant population, and we're going to create more disparity if we just focus on this Blue Cross population, which in our area are prevalently Caucasian…. You know, they're not underinsured in most situations.”
Stakeholder 50 (IHI): “Blue Cross Blue Shield… took a payer blind approach… And I think that also made it a lot easier for… systems to move forward, because they didn't have to worry about doing this only for some patients and not for others. So I think that that helped to remove… a perceived barrier around the scale of what teams could do moving forward.”
Stakeholder 36 (IHI): “The improvement projects don't solely have to focus on Blue Cross Blue Shield patients. They are… payer agnostic, as our health system[s] say. I think it helped with buy‐in….”

ACO, accountable care organization; AIM, acceptability of incentive measure; AQC, Alternative Quality Contract; BCBSMA, Blue Cross Blue Shield of Massachusetts; CMS, Centers for Medicare and Medicaid Services; EHR, electronic health record; EIM, equity incentive measure; HIM, hospital incentive measure; IHI, Institute for Healthcare Improvement; JCAHO, Joint Commission on Accreditation of Healthcare Organizations; NCQA, National Committee for Quality Assurance; PMPM, per member per month; REaL, race, ethnicity, and language; SDOH, social determinant of health; SO/GI, sexual orientation and gender identity.

We've been doing [the AQC] for a very long time and really have seen the benefit from it. So, leveraging that existing structure to then bring this in. … and I know that many others have similar type structures in place. But having that as a basis I think has … been really helpful. (Stakeholder 14)

Based on previous experience of designing the AQC, BCBSMA learned that it was important to link payments for controlling costs to quality (i.e., managing costs of care under a quality constraint). To prevent an exclusive focus on either quality or cost, BCBSMA explicitly linked quality to cost via an efficiency risk‐share arrangement such that for a given physician group, their incentive pay would be dependent not only on meeting or beating their global budget goal (controlling cost growth) but also on their quality performance. To expand this lesson about quality to incorporate equity, BCBSMA developed a linked quality equity score in their incentive payment formula, which was still linked to cost. As a BCBSMA stakeholder explained:

So if you beat your global budget… but had very poor quality, you would only get a small percentage of that surplus paid out to you… as opposed to if you were very high quality, you would get most of it… *So the way we introduce equity into the model was a component of… the quality score*. So making… now a joint quality‐equity score that then also then played into that risk share and linked to the affordability [costs of care]. (Stakeholder 15)

The new incentive formula also includes minimum performance thresholds for quality of care to ensure that improving equity at the expense of overall quality of care was discouraged. Also, participating physician groups who significantly improved quality and equity could earn incentive payments that offset overspending if efficiency targets were not met.

BCBSMA also uses an “equity weight” specific to each physician group designed to ensure that equity improvement is rewarded most when baseline measure denominators among racial and ethnic minority groups are larger and/or baseline inequities are larger. This way, greater equity improvement is rewarded. As noted by a BCBSMA stakeholder:

We considered whether [physician group comparisons]… should be the same for equity [as quality] and eventually decided against that… […] So someone that starts from a very, very bad place can… earn more reward for getting to a level of inequity that's still “worse” than someone else. […] we're… putting… more on the table for poor performers. (Stakeholder 15)

To facilitate buy‐in for equity incentives and reduce the risks of unintended consequences, BCBSMA included only “upside risk” for the equity component of the AQC so that there would be no penalties for worsening equity performance compared with baseline. If a physician group improved overall quality of care but did not improve equity of care by patient race and ethnicity, their lack of equity improvement would not prevent them from earning bonuses for improving overall quality of care. Improving equity of care could also save physician groups money that they would otherwise owe to BCBSMA in the event of having a deficit based on their negotiated global budget, which was already the case for quality performance within BCBSMA's original AQC. Physician groups who successfully improved equity performance but exceeded their global budget would need to repay substantially less money back to BCBSMA compared with if they did not participate. BCBSMA also calibrates payments in such a way that physician groups are paid more for a given combined quality equity score than if the same score had been for quality alone. This incentive design feature was intended to provide physician groups assurance that they would not lose money for failing to improve equity during the initial years of being subject to equity incentives. Without calibration, an equity score of zero would have resulted in lower total payment compared with the original AQC.

BCBSMA provided physician groups with extensive technical assistance to communicate the technical specifications for the equity of care performance measures and how the equity incentive was integrated into the AQC.^9^ Physician group stakeholders, however, did not always fully understand the complex relationship among quality, equity, and cost incentives in the overall performance‐based financial incentive formula. For example, one physician group expressed uncertainty about the indirect impact of equity performance on incentive payments for managing cost growth because the amount that physician groups could earn as a percentage of any budget surplus (i.e., their risk share for efficiency of care) had previously been mediated by their quality performance but was now jointly mediated by both quality and equity performance. As that physician group stakeholder explained:

… It is all feeling quite new, and what I think is going to be a shock to the system is any downside risk…. We're used to upside…. […] In Blue Cross there is a… modifier that relates. Not only do we get a per member per month [PMPM]… incentive payment that's very, you know, you get it or you don't, there's a maximum…. But on the surplus deficit calculation, there is a… modifier that currently is based on the quality score. So if… and when… there's a second modifier that's for equity, that will become very real… and I would just say we're not, haven't really done those equations yet. (stakeholder 25)

Another physician group stakeholder discussed the equity component of the AQC (assessed via internal comparison) and the quality component of the AQC (assessed via external comparison):

So…the incentive program is great in…[the] sense [that]…. […] they're truly looking at our performance and whether or not we've been able to change our own performance. When you look at…the *other components that go into the quality* that's where…at the end of the day, it's whether or not we hit the measures, but those measure thresholds are set on network performance… So at the end of the day, it may not be a direct comparison… but… in…the other ambulatory measures, how our network [physician group] does affects what goals we have to hit. So when our peers in our network [physician] group have been able to improve their [quality] performance year over year, the thresholds have increased and our performance has remained fairly flat… And, you know, it's… trickling up…but we are not able to accelerate at the rate of that network. (Stakeholder 21)

### Alignment of Equity Measure Specifications and Reporting Requirements Across Payers

Key stakeholders indicated that alignment of equity measurement and reporting requirements across commercial and public payers was needed to help efficiently use organizational resources to improve equity. As a physician group stakeholder noted:

[…] There's… a million different data standards out there. […] It drives me a little nuts when payers have different flavors of the same thing. And on something like this, it's… so, so important. Like, probably one of the most important things I will do in my career. It's like, can we just all be aligned so we're on the same plane? (Stakeholder 42)

Participating physician groups serve substantial numbers of patients from other commercial payers and MassHealth, Massachusetts's Medicaid agency. The amounts of money at stake from other payers’ equity incentive programs and data requirements hold considerable weight in physician groups’ long‐term planning and infrastructure investments. As one physician group stakeholder commented:

…MassHealth is really coming up first for us, [and] those [data] requirements are informing what will be our long‐term health equity strategy… […] We don't have final standards … so we're winging it based on draft or preliminary standards…. I think that the hope is… we say: hey, BCBSMA, we've got these standards for MassHealth, we[’ve] got to implement them… we're going live with them, we're making the changes in EPIC [the electronic health record system], please use the same ones… (Stakeholder 42)

Another physician group stakeholder commented: “[…] The [1115] waiver, for health equity…. […] is relative, but it's $350 million annually across all the ACOs [accountable care organizations] that are participating in the Medicaid program here in Massachusetts,” (stakeholder 23).

Those considerations have influenced physician group choices about whether full engagement in BCBSMA's equity initiative components makes financial sense for them. As a BCBSMA stakeholder reported:

[…] One group…. […] opted not to participate in [learning collaborative activities]. And their rationale is like: well, we want to wait and hear what MassHealth is doing, because …we don't want to start something with you and then have to shift it or change it based on what MassHealth requirements are going to be… […] It's unfortunate… they're leaving money on the table. But for them… they see it as the right business call. (Stakeholder 11)

Beyond BCBSMA and MassHealth, physician groups mentioned emerging equity data requirements they need to respond to from the Joint Commission on Accreditation of Healthcare Organizations, the National Committee for Quality Assurance, and Centers for Medicare and Medicaid Services (CMS) reporting requirements for Medicare accountable care organizations (ACOs). Meeting these requirements requires data infrastructure investments, although other payers were not providing funding for data upgrades. Consequently, BCBSMA's equity investments in physician groups are already having positive spillover effects on patients covered by other payers who are competitors in most instances. BCBSMA pilot project grant funds enabled physician groups to upgrade infrastructure for collecting race and ethnicity data and for collecting language, sexual orientation, and gender identity data, social risk data, and disability data, which are included in MassHealth requirements. As a physician group stakeholder explained:

We are… try[ing to]… take advantage of this [BCBSMA] grant funding to build our… health equity infrastructure…We're leveraging what we're doing… with Blue Cross Blue Shield to achieve our contract requirements for MassHealth”; “What we've decided to do is bring all these requirements in one place.… So for care management, for example, I look at what the MassHealth contract requires of them. I look at what the Blue Cross contract requires of them, and I look at… what accreditation requirements are, and then I put them together and give them a package of requirements for which they must build capabilities… And if we have the Blue Cross funding… I can say: here's a pot of money that you can use to build this capability. It's going to fulfill our requirements once we're done. (Stakeholder 44)

Payer‐agnostic investment in equity infrastructure and equity‐focused interventions was a key facilitator of physician group buy‐in to the BCBSMA's incentive program. There was strong physician group stakeholder consensus that BCBSMA's support for payer‐agnostic interventions were central so that physician groups did not exacerbate inequities by diverting their focus and resources to BCBSMA patients and away from non‐BCBSMA patients, who may be more likely to have elevated social risk factors and/or to be racial and ethnic minority patients. For example, as one physician group stakeholder explained:

We're…not just going to take the $2 million and ask the practices to only focus on BCBSMA patients. That would be completely against the whole concept of equity… […] because there's going to be a… sort of a general interest in, well, let me work on that Blue Cross patient roster first. (Stakeholder 23)

### Considering Populations Served, Data and Improvement Infrastructure, and Social Risk Factors

Physician groups serving relatively high proportions of Medicaid populations reported unique challenges of improving equity of care by patient race and ethnicity because of social risk factors (Table 4). Like past experiences of pay‐for‐performance programs not explicitly focused on equity, some physician group stakeholders indicated that improving equity for patients with higher social risk would require more resources than improving equity for patients with lower social risk.^3^, ^21^, ^22^ Some stakeholders worried that there may not actually be “more on the table” for low performers if social risk factors are root causes of poor outcomes for racial and ethnic minority patients. Physician groups participating in MassHealth's ACO payment model correctly noted that ACO payments adjust for social risk factors data, but some stakeholders incorrectly assumed that MassHealth's equity improvement incentives would also adjust for social risk factors and suggested social risk factor adjustment for BCBSMA equity improvement incentives. Even if social risk factor data were consistently available to risk‐adjust payments, adjusting for social risks would make little to no difference because within‐group comparisons are used to calculate incentive payments for equity improvement. If the social risk factor profile of a physician group's patient population drastically changed over time, however, the use of within‐group comparisons may not adequately account for the change.

**Table 4:** Considering Physician Groups’ Populations Served, Data and Improvement Infrastructure, and Social Risk Factors

Physician groups who include safety‐net organizations reported uncertainties in advancing racial equity because social risk factors, including food insecurity, housing instability, and limited transportation may impede equity improvement.
Stakeholder 44 (physician group): “…As part of the contract I think MassHealth is going to provide… We are promised to have… an… upfront health equity… adjustment for people of color, for marginalized groups… We also will have an incentive for meeting the health equity targets… Right now, there's a risk adjustment that is provided. So I think health equity will be treated… like that. There'll be some formula that is applied in… that risk calculation.”
Stakeholder 12 (BCBSMA): “…This is… a big difference between what Blue Cross is doing and what the providers would like the state to do. […] MassHealth is not just hospital visits and doctor visits, there's all the social aspects… […] So providers would want the whole picture of a member… and Blue Cross only has that healthcare part of the member… they were saying… It's just that… as a health plan, we… would never have access to all of that… But the state does.”
Some physician groups stakeholders believed that improving equity for patients with elevated social risk factors requires more resources than improving equity for patients with fewer social risk factors.
Stakeholder 21(physician group): “So…the incentive program is great in…[the] sense [that]…. […] they're truly looking at our performance and whether or not we've been able to change our own performance. When you look at…the other components that go into the quality that's where…at the end of the day, it's whether or not we hit the measures, but those measure thresholds are set on network performance… So at the end of the day, it may not be a direct comparison… but… in…the other ambulatory measures, how our network does affects what goals we have to hit. So when our peers in our network group have been able to improve their performance year over year, the thresholds have increased and our performance has remained fairly flat… And, you know, it's… trickling up. […] but we are not able to accelerate at the rate of that network.”
Safety‐net physician group stakeholders are uncertain about their rate of equity improvement given barriers presented by social risk factors.
Stakeholder 21 (physician group): “[…] I… don't think anyone disagrees with being held accountable to outcomes at some point… But… still… outcomes year one for something as big of a topic as health equity is difficult…. And to be able to make a truly meaningful difference in a single calendar year is… an uphill battle…. […] We may not get any incentive in year one…. […] We're still going to continue doing our work in the clinic and at the system level. But… we… may just not get that incentive as they intend it.”

BCBSMA, Blue Cross Blue Shield of Massachusetts.

Physician group stakeholders generally believed that equity improvement for racial and ethnic minority patients with higher social risk would require more resources than equity improvement for racial and ethnic minority patients with lower social risk. As BCBSMA explained during their annual equity methods retreat for stakeholders, within the equity incentives, there would be “zero payment if [there is] no equity improvement” [of a given physician group, measured against itself, within a given year]. However, the equity incentives are also explicitly designed to “reward improvements that take time to achieve” and should not “penalize providers who serve more diverse populations.”

Even though physician groups are only being compared with themselves for equity improvement, which is viewed as positive, some groups still expected that the equity incentives should be achievable within the first year of the program and expressed concern about the timeframe needed to eventually earn the financial incentives for equity improvement. As a physician group stakeholder explained:

[…] I… don't think anyone disagrees with being held accountable to outcomes at some point… But… outcomes year one for something as big… as health equity is difficult…. And to be able to make a truly meaningful difference in a single calendar year is… an uphill battle…. […] We may not get any incentive in year one…. […] We're still going to continue doing our work…. But… we… may… not get that incentive as they intend it. (Stakeholder 21)

## Discussion

Financial incentives to advance equity of care are an emerging method to encourage health care organizations to simultaneously improve quality, advance equity of care by patient race and ethnicity, and control the escalating costs of care. Our results identified several features of the BCBSMA program seen as important by its designers and physician groups, namely, the design of the performance measures, use of an existing value‐based financial incentive structure that participants were used to, and a reward structure focused on upside risk, improvement within physician groups over time, and protections for safety‐net organizations.

Although physician groups largely reported support for the integration of equity incentives into their value‐based payment arrangements, they raised concerns about the potential for lack of alignment of equity of care performance measures and expectations across payers and the challenge of accounting for social risk factors that contribute to inequity on the quality‐of‐care measures. These findings reflect long‐standing physician group concerns with pay‐for‐performance incentive programs, but they are heightened because system‐level interventions used to improve quality in the past may not be sufficient to improve equity of care by patient race and ethnicity.^3^, ^4^, ^5^ Moreover, even though financial incentives for equity of care performance are designed as bonuses and not penalties, many physician group stakeholders expected that earning rewards in the first year should be achievable. Some physician groups, however, were uncertain about whether they could realistically earn equity incentive payments during the first performance year.

Given the potential for physician groups to improve quality without improving equity of care by patient race and ethnicity, our analyses revealed unique challenges related to comanagement of quality and equity of care. In addition to the perceived high challenge of improving equity of care by patient race and ethnicity without clear evidence‐based interventions, social risk factors were perceived as impediments to achieving incentive payouts. Similar to other pay‐for‐performance programs, key stakeholders were concerned that safety‐net provider organizations, which often have lower overall quality performance, not be further disadvantaged when assessing equity performance.^3^, ^23^ If it is easier for low performers to improve quality and equity and receive their incentives; this evidence could help ease concerns and facilitate future physician group buy‐in. If it is not easier for low performers to improve equity, this could equip health plans with valuable information to consider when accounting for structural differences across physician groups who create barriers to equity improvement.

Although BCBSMA's AQC incentives require minimum performance thresholds for quality of care to ensure that improving equity does not come at the expense of improving overall quality of care, we found that physician group stakeholders were concerned about the implications of this incentive design feature because they do not have historical data to know what to expect in terms of possible equity improvement over time. Moreover, uncertainty about the relative speed at which they could earn equity incentive payments is heightened when equity incentives are integrated into value‐based incentive programs because of their limited experiences of monitoring changes in equity of care. This is unlike value‐based payment programs that reward physician groups based on quality performance and do not consider equity; most groups have past experiences of conducting quality improvement initiatives and therefore understand the level of improvement to expect from these initiatives.^24^ Empirical data about the achievability of simultaneously improving equity and quality and controlling cost growth should be a high priority for health plans and physician groups to collaboratively examine.

To avoid penalizing physicians who serve more disadvantaged patient populations and to reward improvements that take time to achieve, health plans should consider structuring performance‐based equity incentives as BCBSMA has done, including integrating quality and equity scores and basing incentive payouts based on within‐physician‐group comparison over time. The use of within‐group comparisons rather than between‐group comparisons at a cross‐section has the advantage of making risk adjustment less relevant. Most risk‐adjustment models are based on cross‐sectional comparisons, and statistical adjustments are made to assist with making fair comparisons among physician groups.^25^ Because physician groups are not explicitly being compared with one another for the purpose of determining equity incentives, risk adjustment is less relevant within the context of BCBSMA's equity initiative. Our analyses, however, indicate that some physician group stakeholders were still concerned about the need to adequately risk‐adjust equity of care performance despite BCBSMA's use of within‐physician‐group comparisons for determining equity incentives.

We found that physician group stakeholders serving high shares of MassHealth members preferred social risk factor adjusted equity performance measures over unadjusted measures, but social risk factor data are limited and there is considerable debate about whether adjusting for social risk factors promotes or hinders equity improvement.^26^ Our analyses also revealed that some physician group stakeholders sometimes did not understand how social risk factors were incorporated into payment and incentive methods across payers. For example, MassHealth's ACO risk‐adjustment method for per‐member‐per‐month (PMPM) payment are adjusted for social risk factors, but their planned equity improvement incentives as part of their 1115 waiver will not be.^26^ PMPM adjustment, as used in CMS's ACO REACH model, entails establishing a multiplier so that physicians serving a more socially at‐risk population are rewarded more for achieving the same level of equity improvement as that of their peers serving a more advantaged population.^27^

Although prospective adjustment for social risk factors could best support physician groups, doing so is more challenging for commercial health plans, which need to negotiate payment rates with local physician groups who often have high market power.^28^, ^29^ Comparing the impact of different value‐based incentive payment methods, including social risk factor risk‐adjusted PMPM with and without equity incentives, on equity improvement could provide insight on how to best change physician group behavior to address enduring racial and ethnic disparities in quality of care.

Taken together, our results underscore that even with carefully designed equity incentives, substantial BCBSMA upfront infrastructure support, and technical assistance to help physician groups understand equity measures and incentives, physician group stakeholders were sometimes uncertain about the adequacy of protections for safety‐net organizations serving higher shares of populations with elevated social risk factors. Within‐group comparisons for determining equity incentive payments has many advantages, but empirical evidence is still needed to clarify the extent to which the social risk profiles of physician groups are stable over short time periods and not inhibiting equity improvement. This evidence could also ease some stakeholder concerns about the potentially confounding role of social risk factors on equity improvement.

It is important to note that unlike other value‐based payment initiatives, common challenges related to methods for attributing BCBSMA patients to physician groups and managing patient denominator changes by performance measure were well established prior to the introduction of the equity incentives into the AQC. Although differential attrition of patients by race and ethnicity over time did not surface as an equity concern in our analyses, past evidence indicates that insurance instability can impact quality of care among racial and ethnic minorities.^30^ Given this, the impact of insurance instability on equity of care performance should be monitored as equity incentives are integrated into value‐based incentive programs.

Our study has some noteworthy limitations. First, physician groups were at different stages of contract negotiations when interviews were conducted, so not all contracts were finalized. This could be another reason besides equity incentive design complexities that accounts for physician group uncertainties about the incentives. Second, although we had a high response rate for the interviews, we did not capture the experiences of all participating physician groups. Third, we did not consistently interview multiple key informants from each group, so within‐group reliability could not be assessed. Finally, the results do not generalize to physician organizations not yet receiving value‐based payments. In 2022, however, 46% of primary care physicians provided care in practices receiving value‐based payments.^31^ Our study results are relevant to a growing number of health plans and physician organizations that are considering integrating of equity of care performance measures and equity incentives into their value‐based payment programs when physician group contracts are renewed, and value‐based payment incentive requirements are revised.

### Conclusion

Our analyses of the launching of one of the nation's first large‐scale, performance‐based financial incentives to advance equity of care by patient race and ethnicity revealed the importance of valid and reliable equity performance measurement and the need for major health plan and physician group investments to improve the completeness and accuracy of patient race and ethnicity data. Empirical evidence indicates that the most promising equity‐focused interventions are culturally tailored to meet patients’ needs and use interprofessional team approaches, interactive techniques to deliver self‐management education, and family and/or community member involvement, which could require investment by all payers.^17^, ^18^, ^19^ Given the major data infrastructure investments and organizational change management resources required of physician groups to prepare for equity incentives and implement equity‐focused interventions, aligning requirements across commercial and government payers could facilitate groups’ efforts to improve equity of care by patient race and ethnicity.

## Funding/Support

This work was funded by The Commonwealth Fund (20222738).

## Conflict of Interest Disclosures

The authors report no relationship or financial interest with any entity that would pose a conflict of interest with the subject matter of this article.

## Supporting information



Appendix 1‐4
