# Supplementary material for: Launching Financial Incentives for Physician Groups to Improve Equity of Care by Patient Race and Ethnicity
Source: Milbank Q. 2024 Oct 25;102(4):944–72. doi: 10.1111/1468-0009.12720 (PMC11654755; doi:10.1111/1468-0009.12720)
Supplement: Supplementary file 1 — Appendix 1‐4 [file MILQ-102-944-s001.docx]

**Appendix 1**

**Alternative Quality Contract Participants**

**At the Beginning of the Interview**

Did you get a chance to review the consent document, and do you have any questions?

- If yes, no questions—“Great! Do I have your consent to begin recording?”
- If no—quickly review key points:
  - We are from the University of California, Berkeley, conducting research to better understand the Blue Cross Blue Shield of Massachusetts (BCBSMA) equity initiatives.
  - This interview will be approximately 45 minutes, but you can end it at any time. We will contact you in about a year to participate in a second interview.
  - The interview will be deidentified, and nothing you say will be attributed to you.

We would like to record this interview to ensure we capture it accurately. Do I have your permission to record?

*Wave 1*

- 1. Could you tell us about your role and how you became involved in the equity initiatives with BCBSMA?
  2. Which parts of your organization are working on BCBSMA equity initiatives?
- Probes: departments/levels of hierarchy/professions
- Fit with other internal equity efforts, or work with other payers?
  1. Which quality, access, and patient experience measures are the focus of your group’s strategies to advance health equity?
- Why and how were these measures prioritized?
- Any measures/priorities for health equity outside the Blue Cross pay-for-equity measures?
- How did your group decide on what to propose for the Institute for Healthcare Improvement equity grant?
- What work will you be doing with that grant?
- How do you plan to sustain these investments over time?
- How familiar are you with Blue Cross pay-for-equity design?
- Strengths/weaknesses from your perspective?
- Within your group, what have discussions about the Alternative Quality Contract’s “pay for equity” incentives been like?
- Is there a feeling that progress on the equity goals is achievable?

1. Probe if needed: Does the expectation of achievability vary by performance measure/target (e.g., hypertension control, A1c control, breast cancer screening, and adolescent well visits)?

- What resources or organizational changes will be needed to make progress on equity measures?
- How have customized health equity reports produced by BCBSMA been used (if at all) by group leaders?
- Discussed in any meetings? Shared with different departments?
- Which aspects were new/surprising vs. already known from internal data?
- Any actions taken after reviewing the report?
- How can they be improved?
- Which parts of the group’s work on equity with BCBSMA (measurement, strategies for improvement) are being applied uniformly across your group? Which parts are being done differently across practice locations?
- How have practice leaders and frontline clinicians reacted to the health equity measures? What kinds of concerns have people raised?
- Did you participate in any “pay-for-equity” design and feedback sessions with BCBSMA? If so, can you tell us how that went?
- Perspective on ideas that were especially promising?
- Concerns about unintended consequences?
- Incentive design and participation requirements?
- How does your work with BCBSMA on equity fit into the other strategic priorities of your group? Like financial goals, quality goals, other equity initiatives? Pressures or initiatives from other payers?
- To what extent does your group expect a financial return on your investments to advance health equity?
- To what extent does your group *currently* collect information about social risk factors, such as food insecurity, housing instability, and/or transportation needs and address these nonmedical barriers as part of chronic care management programs?
- Are Z-codes used to document social risks? Or another system? Or both?
- Please describe your group’s future plans to address social risk factors as part of your efforts to reduce inequity.

**Appendix 2**

**Blue Cross Blue Shield of Massachusetts Equity Initiatives: Program Manager/Internal Stakeholder Interviews: Interview Questions**

**At the Beginning of the Interview**

Did you get a chance to review the consent document, and do you have any questions?

- If yes, no questions—“Great! Do I have your consent to begin recording?”
- If no—quickly review key points:
  - We are from the University of California (UC), Berkeley, conducting research to better understand the Blue Cross Blue Shield of Massachusetts (BCBSMA) equity initiatives.
  - This interview will be approximately 45 minutes, but you can end it at any time.
  - The interview will be deidentified, and nothing you say will be attributed to you.
  - We would like to record this interview to ensure we capture it accurately. Do I have your permission to record?

*Wave 1*

- - 1. From your understanding, could you tell us what you consider to be the key components of BCBSMA’s health equity initiatives? Probe: Have what are considered the key components changed over time, and if so, why?
    2. Thinking about the different provider groups, what factors have you noticed so far that could help some groups be more successful than others? Any factors that could make it difficult for certain groups to achieve their targets? Probe: Has this changed over time, and if so, why?
    3. How, if at all, are socioeconomic differences accounted for when measuring racial and ethnic disparities within and between groups? Probe: Is there any pressure to look at additional measures of disparity in addition to or instead of race and ethnicity?
    4. How long do you expect it to take for provider groups to achieve their equity targets? Probe: Has this changed as your team has learned about equity improvement (or lack thereof) reported by groups or in BCBSMA’s claims data?
    5. Why do you think it will take x amount of time to meet the equity targets? Probe: Have these reasons changed over time, and if so, why?
    6. To what extent did the provider groups use your team’s expertise to achieve their goals? How about Institute for Healthcare Improvement (IHI) coaches?
    7. How will the relationship with IHI look moving forward? Probe: Any challenges of coordination with IHI?
    8. What changes on BCBSMA’s health equity team have been made to take on some of the work currently being done by IHI?
    9. Are there new organizational partners involved in BCBSMA’s equity initiatives, and how did they contribute? Probe: Have equity initiative partnerships changed over time, including the discontinuation of partnerships?
    10. Which employer customers have requested health equity data for their employees within the last year? How did they respond and use these data, if at all? Probe: How has this evolved over time, and if so, why?
    11. What is your sense of provider group’s perception about the impact of their equity investments, including data infrastructure and targeted interventions?
    12. What policy changes and/or resources do you think that provider groups will be needed to sustain their progress on advancing equity in quality of care with the foundation of BCBSMA’s support?

**At the Beginning of the Interview**

Did you get a chance to review the consent document, and do you have any questions?

- If yes, no questions—“Great! Do I have your consent to begin recording?”
- If no—quickly review key points:
  - We are from the UC, Berkeley, conducting research to better understand the BCBSMA equity initiatives.
  - This interview will be approximately 45 minutes, but you can end it at any time. We will contact you in about a year to participate in a second interview.
  - The interview will be deidentified, and nothing you say will be attributed to you.
  - We would like to record this interview to ensure we capture it accurately. Do I have your permission to record?

**Institute for Healthcare Improvement Stakeholders**

- - 1. Could you tell us about your role and how you became involved in the health equity initiative with BCBSMA?
    2. Can you walk me through the processes that occurred as part of the learning collaborative—meetings, analysis, technical assistance, etc. Any changes made to plans for the collaborative along the way?
- Any unique aspects for this collaborative relative to IHI standard practice?
  - 1. What kinds of goals did the Alternative Quality Contract (AQC) groups work on in the learning collaborative (e.g., culturally tailored interventions such as diabetes self-management education programs in Spanish for Latinx populations, barber shop interventions for Black men, data systems)?
- How much did AQC groups vary on priorities? Which roles were represented (level of seniority, clinical/nonclinical backgrounds)? Their preparation/commitment?
  - 1. What elements of the collaborative went well/met intended goals from your perspective? What if any goals were not met?
- What changes are being planned or implemented to address unmet goals?
  - 1. Thinking about the different provider groups, what factors have you noticed that could help some groups be successful? Any factors that could make it difficult for certain groups to achieve their targets?
- Any interventions that seemed to be especially challenging/likely to be abandoned?
- What made these interventions challenging?
  - 1. What types of potential unintended consequences came up in the learning collaborative? For example, AQC group “gaming” of racial disparities measures (excluding racial minority patients from services and/or from the data, not collecting race and ethnicity data for groups perceived to present barriers to achieving targets)?
- What has been done, if anything, to address gaming?
- So far, what would you say are the most important learnings from this experience that *other payers and other states* should know about advancing pay for equity?

**Appendix 3**

**Health Equity Council Members**

**At the Beginning of the Interview**

Did you get a chance to review the consent document, and do you have any questions?

- If yes, no questions—“Great! Do I have your consent to begin recording?”
- If no—quickly review key points:
- We are from the University of California, Berkeley, conducting research to better understand the Blue Cross Blue Shield of Massachusetts (BCBSMA) equity initiatives.
- This interview will be approximately 45 minutes, but you can end it at any time. We will contact you in about a year to participate in a second interview.
- The interview will be deidentified, and nothing you say will be attributed to you.

We would like to record this interview to ensure we capture it accurately. Do I have your permission to record?

- - 1. Could you tell us about your role and how you became involved in the Health Equity Council with BCBSMA?
    2. From your perspective, how did the comprehensive strategy to advance health equity emerge within BCBSMA? Were there any key milestones or decision points?
    3. Could you tell us what you consider to be the most important components of the health equity initiative?
    4. What role has the Health Equity Council played in shaping, planning, and implementation of the initiative? Have you seen examples of BCBSMA adapting approaches based on the Health Equity Council’s feedback? Probe for specifics:
- pay for equity as part of the Alternative Quality Contract (AQC)
- Institute for Healthcare Improvement learning collaborative
- grants to AQC groups to support their infrastructure and interventions to advance racial equity
- vendor diversity efforts
- race and ethnicity/language data collection from members

1. Are there any examples in which BCBSMA’s planning or implementation has gone in a different direction from recommendations raised by the Health Equity Council?

- Why was a different direction pursued?
- How do you think that these decisions will affect the initiative?

1. Have there been any significant changes to the AQC measure or incentive design so far? Probes:

- key events
- stakeholders
- data that prompted changes
  - 1. Thinking about the different provider groups, what factors have you noticed so far that could help some groups be more successful than others? Any factors that could make it difficult for certain groups to achieve their targets?
- Any interventions that seemed to be especially challenging/likely to be abandoned?
- In your perspective, what made these interventions challenging?
- What changes should be made to these interventions?
  - 1. To what extent are there concerns about AQC group “gaming” of racial disparities measures? For example, excluding racial minority patients from services and/or from the data, not collecting race and ethnicity data for groups perceived to present barriers to achieving targets or creating barriers to care. Concerns about other unintended consequences?
- Do you think some people may be reclassified racially or ethnically to alter results?
- Have you noticed if any effort has been made to reduce gaming by BCBSMA and/or participating AQC group leadership?
- What role can the health equity council take to address these issues?
  - 1. Do you anticipate that BCBSMA’s pay-for-equity component will reduce racial inequity in Healthcare Effectiveness Data and Information Set measures as part of the AQC, for example, reduced racial disparities in hypertension control, A1c control, breast cancer screening, and adolescent well visits? Why or why not?

**Appendix 4**

**Blue Cross Blue Shield of Massachusetts Equity Initiatives: Executive Interview Questions**

**At the Beginning of the Interview**

Did you get a chance to review the consent document, and do you have any questions?

- If yes, no questions—“Great! Do I have your consent to begin recording?”
- If no—quickly review key points:
  - We are from the University of California, Berkeley, conducting research to better understand the Blue Cross Blue Shield of Massachusetts (BCBSMA) equity initiatives.
  - This interview will be approximately 45 minutes, but you can end it at any time. We will contact you in about a year to participate in a second interview.
  - The interview will be deidentified, and nothing you say will be attributed to you.
  - We would like to record this interview to ensure we capture it accurately. Do I have your permission to record?

*Wave 1*

- - 1. Could you tell us about your role in the organization and how you became involved in equity initiatives?
    2. How did the comprehensive strategy to advance health equity emerge within the organization?
       1. Key milestones or decision points?
       2. Were some aspects of equity initiatives easier to get agreed upon than others?
    3. Could you tell us what you consider to be the key components of the health equity initiative?
    4. Have there been any significant changes to the Alternative Quality Contract (AQC) measures or incentive design so far? Probes: key events, stakeholders, data that prompted changes?
    5. How have contract negotiations gone? Sticking points?
    6. How, if at all, are socioeconomic differences accounted for when measuring racial and ethnic disparities within and between groups?
    7. To what extent are there concerns about AQC group “gaming” of racial disparities measures? For example, excluding racial minority patients from services and/or from the data, not collecting race and ethnicity data for groups perceived to present barriers to achieving targets.
    8. What internal investments do you anticipate groups will need to make, beyond the Institute for Healthcare Improvement grants awarded, in order to achieve the new pay-for-equity targets?
    9. How long do you expect it to take for groups to achieve their equity targets?
    10. So far, what would you say are the most important learnings from the BCBSMA experience that other payers and other states should know about advancing pay for equity?
    11. Which employer customers took up the option of receiving health equity data for their employees? How did they respond and use these data, if at all?
    12. What is the perception about the sustainability of the changes made? What resources will be needed to sustain the progress made with BCBSMA’s support?
